# Physiological Indicators for Post-Translocation Monitoring of *Salix lapponum* in Natural vs. Degraded Peatlands

**DOI:** 10.3390/plants15101557

**Published:** 2026-05-20

**Authors:** Michał Arciszewski, Magdalena Pogorzelec

**Affiliations:** Department of Hydrobiology and Protection of Ecosystems, University of Life Sciences in Lublin, Dobrzańskiego 37, 20-262 Lublin, Poland; michal.arciszewski@up.edu.pl

**Keywords:** active conservation, biomarkers, chlorophyll fluorescence, downy willow, environmental stress, glacial relics, peatlands, translocation success

## Abstract

The progressive degradation of natural habitats, driven by anthropogenic pressures and climate change, constitutes one of the most serious threats to biodiversity. Peatland ecosystems, along with the valuable plant species associated with them, are particularly vulnerable to these processes. *Salix lapponum*, a glacial relict species, is undergoing a drastic decline in both its range and population size across Poland and Europe. This emphasizes the need for the implementation of conservation measures, including species translocation, as well as the development of effective methods for monitoring plant condition following introduction. The aim of this study was to evaluate the usefulness of selected physiological indicators for the rapid and reliable assessment of plant condition in active conservation efforts. The experimental material consisted of *S. lapponum* plantlets derived from tissue culture, which were introduced into five experimental sites in eastern Poland, differing in habitat conditions. Over two growing seasons, chlorophyll fluorescence parameters (F_0_, F_m_, F_v_/F_m_), the content of photosynthetic pigments and anthocyanins, relative water content, guaiacol peroxidase activity, and the presence of reactive oxygen species were analyzed. The results revealed clear seasonal variability in most of the studied physiological parameters, as well as their differentiation across habitat conditions. The highest sensitivity to environmental changes was observed for indicators related to photosynthetic performance (F_v_/F_m_), tissue hydration status (RWC), and enzymatic activity. Declines in photosystem II efficiency at the beginning of the growing season, reflected in F_v_/F_m_ values decreasing to 0.47–0.49 indicate transient stress conditions in plants. Simultaneously, variation in relative water content (52–90%) and peroxidase activity reflects differences in water availability and the intensity of environmental stress across habitats. The findings confirm that selected physiological indicators can serve as effective tools for the early monitoring of plant condition and for assessing the success of *S. lapponum* translocation.

## 1. Introduction

As a result of the increasing transformation of natural habitats driven by human activity and climate change, environmental conditions in which populations of many plant species persist are undergoing significant alteration [1]. As demonstrated by Staudt et al. [2], the disruption of ecosystem functioning may result from factors such as landscape fragmentation, alterations in water regimes, eutrophication, heavy metal pollution, accelerated ecological succession, and biological invasions. Peatlands are among the habitats that have undergone particularly significant transformation in recent decades. Despite their relatively small global coverage, their degradation leads to disproportionately large ecological losses, making them one of the most threatened ecosystems [3,4,5]. Plants in transformed habitats are exposed to complex environmental stressors, which significantly affect their long-term condition. The primary effects of stress include reduced photosynthetic efficiency, growth inhibition, and disturbances in reproduction, which may ultimately lead to population extinction [6,7].

Glacial relict species are particularly sensitive to ongoing changes in natural habitats. These species persist as remnants of the glacial era and occupy small, isolated climatic refugia, primarily located in mountain and peatland ecosystems. The small population sizes of relict species, combined with their specific habitat requirements, make them especially vulnerable to environmental changes and local extinction [8,9]. Among such species is *Salix lapponum*, whose population size is declining drastically. This species was once widespread across numerous sites in Poland and Europe, but is currently threatened with extinction. *S. lapponum* is a small shrub that occurs in fens and transitional mires with a moderately acidic pH (4 to 6.5) in both open and partially shaded habitats. The species grows up to 1–1.5 m in height and is characterized by elliptical, olive-green leaves covered with a dense layer of tomentum [10,11,12,13]. Research conducted in eastern Poland in habitats occupied by *S. lapponum* has shown that the species most frequently occurs in phytocoenoses representing the classes *Alnetea glutinosae*, *Oxycocco-Sphagnetea*, and *Scheuchzerio-Caricetea* [14]. In Poland, the species reaches the southern limit of its distribution range and is currently found only at a few locations, which has resulted in its classification as critically endangered. The decline in *S. lapponum* populations is primarily driven by habitat changes associated with the drainage of wetlands [15]. In order to protect populations of *S. lapponum* in Poland, reintroduction efforts have been undertaken at sites where the species had previously been recorded [16,17].

In order to protect valuable plant taxa at risk of extinction, species translocations are increasingly used as a method of active protection [18]. However, the results of plant translocations remain highly uncertain because their success depends on many interacting factors, including the selection of an appropriate target site, the quality and origin of the propagation material, the biology and ecology of the species, and appropriate post-translocation monitoring methods. Numerous studies indicate that plant translocation is a process that involves significant expenses and requires careful planning, technical preparation, and effective logistical coordination [19,20,21]. A recent study of translocations in Europe showed that almost half of the applications were lost after five years, and only 28% became self-sustaining. The early mortality was high, with 40% of the effects resulting in less than 20% survival after the first year [22].

The restoration of endangered species populations should be closely linked with the protection of the habitats in which active conservation measures are implemented. Otherwise, the risk of failure of such efforts increases significantly [23]. The instability and ongoing transformation of habitat conditions require verification of whether a given species is capable of surviving under rapidly changing environmental conditions. It is also essential to identify alternative habitats in which the species could find a suitable ecological niche, thereby enabling effective population recovery in new locations [24]. One of the key determinants of successful translocation is the ability of plants to complete their full life cycle, including growth, flowering, and the production of viable offspring [23]. However, such assessment is a long-term process, often requiring many years of field observations. Therefore, there is a need to identify additional, more rapidly responding indicators, particularly physiological and morphological ones, that would enable the early assessment of plant condition and the prediction of translocation success [25]. In the case of plant species threatened with extinction, the development of physiological monitoring protocols requires not only methodological sensitivity but also consideration of nature conservation constraints. Small and fragmented populations often limit the possibility of extensive tissue sampling, making the level of invasiveness an important factor in the selection of monitoring tools. Therefore, conservation physiology is increasingly emphasizing the development of minimally invasive methods that provide reliable information while minimizing disturbance to both individuals and their natural habitats [26,27].

Previous studies on *Salix lapponum* have primarily focused on its ecology, habitat requirements, and the processes driving population decline, particularly in relation to peatland transformation [15]. Experimental conservation efforts have addressed ex situ propagation and reintroduction, and the effectiveness of these actions was mainly evaluated using plant survival rates and morphological traits [17]. More recent research has incorporated physiological parameters, including chlorophyll fluorescence, photosynthetic pigments, and ROS accumulation, but these have been applied mainly under controlled conditions or during early stages of acclimatization. Notably, Pogorzelec et al. [16] analyzed these indicators across successive stages of the reintroduction process, focusing on plant acclimation under conditions corresponding to suitable, reference habitats.

However, the use of physiological indicators for post-translocation monitoring under natural, heterogeneous field conditions remains limited. In particular, there is a lack of studies assessing plant responses across habitats representing different degrees of peatland transformation. Therefore, this study introduces a novel approach by applying a set of physiological biomarkers to monitor *S. lapponum* after translocation across a gradient of peatland habitats differing in transformation level. This experimental design reflects potential future scenarios of habitat change and enables the evaluation of plant responses under realistic, multifactorial environmental conditions.

The aim of this study was to evaluate the usefulness of physiological indicators as tools for monitoring the condition of *S. lapponum* individuals in active conservation using translocation. Additionally, we assessed which of the examined parameters allow for a rapid and reliable evaluation of the effectiveness of conservation measures.

## 2. Results

### 2.1. Selected Chlorophyll Fluorescence Parameters

**Figure 1:**
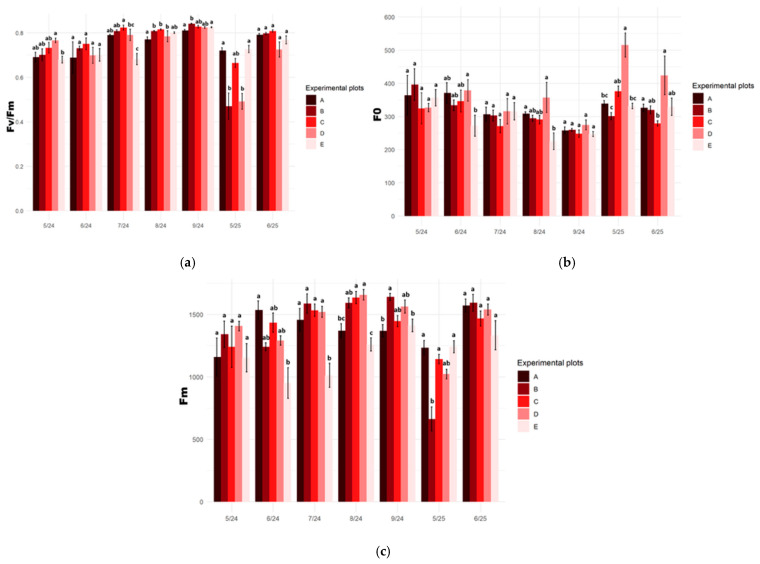
Chlorophyll fluorescence parameters in the leaves of *Salix lapponum* at five experimental sites (A–E) from May 2024 to June 2025: (**a**) maximum quantum efficiency of PSII (F_v_/F_m_); (**b**) minimal fluorescence (F_0_); (**c**) maximal fluorescence (F_m_). Bars represent mean values ± SD, *n* = 10. Different letters above the bars indicate significant differences among sites within a given sampling term (Kruskal–Wallis test, *p* ≤ 0.05). Experimental plots: A—transitional mire (reference site), B—raised bog, C—transitional mire undergoing ecological succession, D—drained peatland, E—degraded peatland.

In 2024, the maximum quantum efficiency of photosystem II (F_v_/F_m_) in plants at the experimental sites increased from May to September, reaching the highest values at the end of the growing season (0.81–0.84). During the first year of the study, F_v_/F_m_ values did not differ significantly among most translocation sites. Only plants growing on the degraded peatland exhibited lower values (approx. 0.69). Clear differences among plants growing in the studied habitats were observed at the beginning of the following growing season (May 2025). The lowest F_v_/F_m_ values (approx. 0.47–0.49) were recorded in plants introduced into the raised bog and the drained peatland. In the remaining habitats, the maximum PSII efficiency values were lower than those recorded at the end of the previous growing season, but similar to those observed during the same period in the first year of the study (Figure 1a).

For the remaining chlorophyll fluorescence parameters (F_0_ and F_m_), only minor statistical differences were observed among plants across the studied sites. Plants growing at the reference site (A) and at the raised bog site (B) were characterized by relatively stable values of both minimal and maximal fluorescence. The lowest values of these parameters, along with pronounced seasonal fluctuations, were recorded in plants inhabiting the degraded peatland site (Figure 1b,c).

### 2.2. Photosynthetic Pigment Content

**Figure 2:**
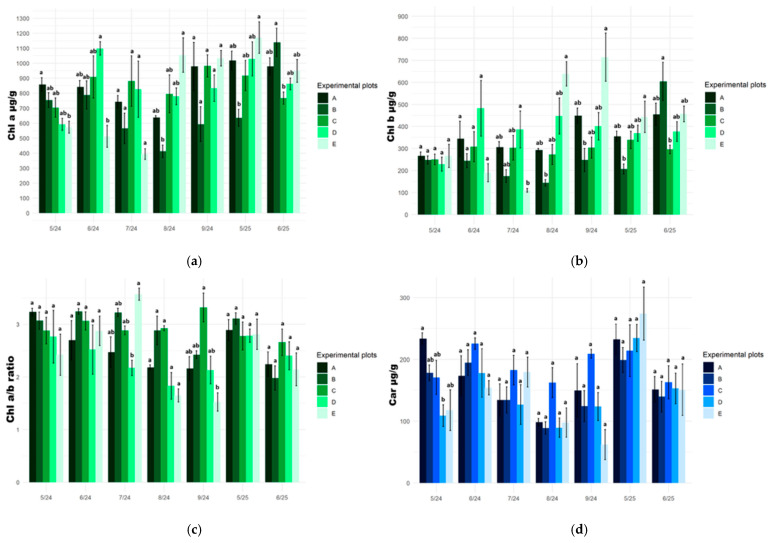
Photosynthetic pigment content in leaves of *Salix lapponum* at experimental sites in consecutive sampling periods (May–September 2024; May–June 2025): (**a**) chlorophyll *a* (Chl *a*; µg·g^−1^ FW); (**b**) chlorophyll *b* (Chl *b*; µg·g^−1^ FW); (**c**) chlorophyll a/b ratio (Chl a/b); (**d**) carotenoids (Car; µg·g^−1^ FW). Bars represent means ± SD, *n* = 3. Different letters above the bars indicate statistically significant differences among sites within a given sampling term (Kruskal–Wallis test, *p* ≤ 0.05). Experimental plots: A—transitional mire (reference site), B—raised bog, C—transitional mire undergoing ecological succession, D—drained peatland, E—degraded peatland.

The chlorophyll *a* (Chl *a*) and chlorophyll *b* (Chl *b*) content in the tissues of *S. lapponum* plants growing at the reference site (A) showed relative stability during both growing seasons. Seasonal variation of the tested pigments in plant tissues was moderate, without sudden fluctuations. Compared to the reference site, the Chl *a* and Chl *b* content in plants growing in the other habitats revealed greater variability, particularly in the degraded peatland (E), where marked decreases and increases in the content of these pigments were observed (Figure 2a,b).

The Chl a/b ratio in the tissues of the studied plants ranged from approximately 1.5 to 3.5 and exhibited significant seasonal variation. In 2024, the highest values of this parameter were recorded in May (site A) and July (site E), whereas in August and September, a decline in the ratio was observed, particularly pronounced in plants at site E. At the beginning of the following growing season (May 2025), the Chl a/b ratio in plant tissues exceeded 2.5 at all sites (Figure 2c).

The carotenoid (Car) content in the tissues of plants introduced into the experimental sites varied dynamically throughout the growing season. However, no statistically significant differences in the carotenoid content in the tissues of *S. lapponum* individuals were observed within the individual sampling times (Figure 2d).

### 2.3. Anthocyanin Content

**Figure 3:**
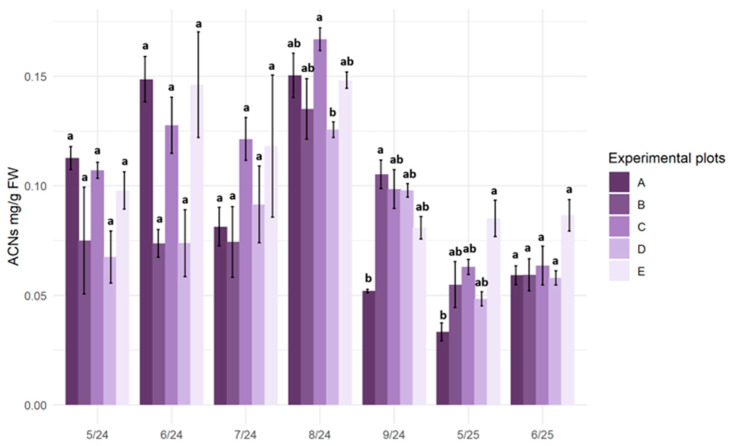
Anthocyanin content (ACNs; mg·g^−1^ FW) in the leaves of *Salix lapponum* at five experimental sites (A–E) from May 2024 to June 2025. Bars represent mean values ± SD, *n* = 3. Different letters above the bars indicate significant differences among sites within a given sampling term (Kruskal–Wallis test, *p* ≤ 0.05). Experimental plots: A—transitional mire (reference site), B—raised bog, C—transitional mire undergoing ecological succession, D—drained peatland, E—degraded peatland.

During the 2024 growing season, the anthocyanin (ACN) content in plant material increased at all translocation sites, with the highest values recorded in August (approximately 0.15–0.17 mg·g^−1^ FW). In September, there was a decrease in anthocyanin content in plant tissues, particularly pronounced at the reference site (A). In the spring of 2025, the anthocyanin content in the studied plants was significantly lower compared to the first year of the study and was lowest in plants growing on the transitional mire (A—below 0.05 mg·g^−1^ FW). At most sampling times, no statistically significant differences in anthocyanin content in the plant material were observed among the plots (Figure 3).

### 2.4. Relative Water Content

**Figure 4:**
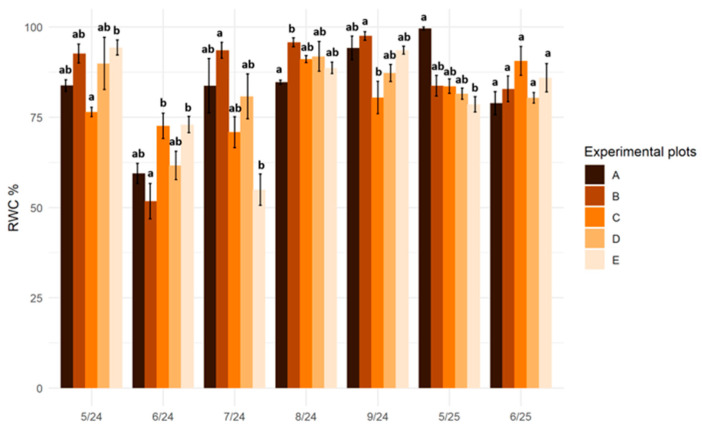
Relative water content (RWC; %) in the leaves of *Salix lapponum* at experimental sites in consecutive sampling periods (May–September 2024; May–June 2025). Bars represent means ± SD, *n* = 5. Different letters above the bars indicate statistically significant differences among sites within a given sampling term (Kruskal–Wallis test, *p* ≤ 0.05). Experimental plots: A—transitional mire (reference site), B—raised bog, C—transitional mire undergoing ecological succession, D—drained peatland, E—degraded peatland.

The relative water content (RWC) in the leaves of *Salix lapponum* exhibited significant variation both across seasons and among sites. During the 2024 growing season, from May to July, the relative water content in plant tissues fluctuated at all experimental sites and reached its lowest values in June (RWC = 52% in plants at the raised bog site—B). In the following months, this value increased significantly in plants, and in most cases, was approximately 90%. In 2025, RWC in the *S. lapponum* tissues remained at a relatively stable, high level. Particularly high values were recorded in May 2025 at the reference site (Figure 4).

### 2.5. Guaiacol Peroxidase Activity

**Figure 5:**
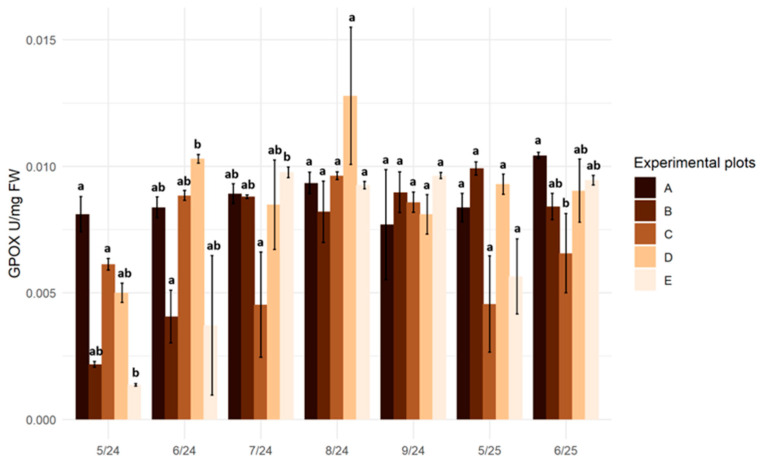
Guaiacol peroxidase activity (GPOX; U·mg^−1^ FW) in the leaves of *Salix lapponum* at experimental sites in consecutive sampling periods (May–September 2024; May–June 2025). Bars represent means ± SD, *n* = 3. Different letters above the bars indicate statistically significant differences among sites within a given sampling term (Kruskal–Wallis test, *p* ≤ 0.05). Experimental plots: A—transitional mire (reference site), B—raised bog, C—transitional mire undergoing ecological succession, D—drained peatland, E—degraded peatland.

At the reference site (A), located in a transitional mire, guaiacol peroxidase (GPOX) activity in plant tissues remained relatively stable throughout the experiment (0.008–0.010 U·mg^−1^ FW). At the raised bog site, a significant increase in enzymatic activity was recorded in May 2025 compared to the same period in 2024. At the remaining sites, guaiacol peroxidase activity in the plant tissues exhibited considerable seasonal fluctuations during the 2024 growing season. Significant differences were observed in plants from the drained peatland (D) and the degraded peatland (E), where activity was lowest in May 2024 and increased during the summer months (Figure 5).

### 2.6. Histochemical Detection of Reactive Oxygen Species (ROS)

**Figure 6:**
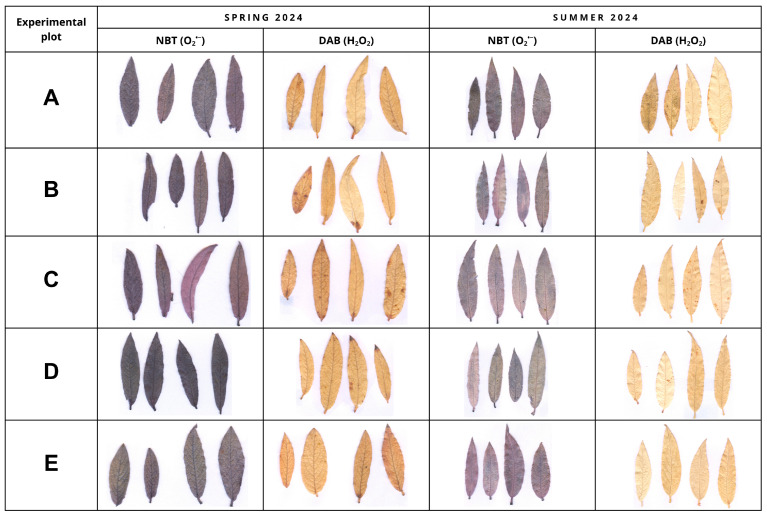
Visualization of the histochemical detection of superoxide anion (O_2_^•−^, NBT staining) and hydrogen peroxide (H_2_O_2_, DAB staining) in *Salix lapponum* leaves collected from five peatland habitats in spring and summer 2024. Experimental plots: A—transitional mire (reference site), B—raised bog, C—transitional mire undergoing ecological succession, D—drained peatland, E—degraded peatland.

Histochemical staining using NBT and DAB revealed the presence of hydrogen peroxide and superoxide anions in plant tissues at all analyzed plots, both in the spring and summer of 2024. Staining intensity varied mainly between seasons and moderately between sites. In spring, significantly more intense tissue staining was observed at all experimental sites for both DAB and NBT reactions compared to the summer period. The most pronounced differences among plants were observed in transformed habitats (D and E), where more extensive and intense staining of the leaf blade occurred, particularly in the NBT reaction (Figure 6).

## 3. Discussion

The transformation and degradation of natural habitats lead to a decline in biodiversity at all levels. The extinction of entire plant populations or a drastic decline in their abundance is often associated with a reduction in the area of habitats to which species are strongly associated, due to their specific ecological requirements [28,29,30]. In such cases, active conservation measures involve not only increasing the population size, but above all, identifying suitable substitute habitats that enable successful establishment, acclimatization, and long-term persistence of self-sustaining populations. On the other hand, it is important to determine whether and to what extent plants can respond to environmental stresses that could potentially affect them under the conditions of new habitats [23,31,32].

The assessment of selected physiological parameters during the experimental translocation of *Salix lapponum* in 2024–2025 enabled the identification of reliable indicators for monitoring plant condition at the early stage of acclimatization in substitute habitats.

The efficiency of the photosynthetic process is determined by prevailing habitat conditions, particularly by light and water availability. A deficiency or excess of either of these factors can cause environmental stress [33,34,35]. Therefore, in degraded habitats or those exposed to frequent fluctuations in abiotic conditions, introduced plants show a greater susceptibility to disruptions in physiological processes.

Chlorophyll fluorescence analysis is a widely used, non-invasive method for assessing the functional status of the photosynthetic apparatus in plants. It enables the early detection of stress responses prior to the appearance of visible morphological symptoms. Maximum photochemical efficiency of photosystem II (F_v_/F_m_) values above 0.75 in dark-adapted plants generally indicate proper photosynthetic performance, whereas lower values may reflect oxidative stress and damage to the photosynthetic apparatus [25,36,37].

The results of chlorophyll fluorescence measurements conducted during the field experiment indicate that the F_v_/F_m_ parameter was the most sensitive diagnostic tool for assessing the condition of *S. lapponum*. Analysis of F_v_/F_m_ values in plants from substitute habitats revealed habitat-dependent variation in physiological responses. It also demonstrated a pronounced stress response in all studied introduced plants in May 2025.

The potential usefulness of this indicator for monitoring the condition of plants introduced to new habitats is supported by studies on other species that require active conservation worldwide [38,39,40]. Research conducted by Liu et al. [39,41] on the critically endangered species *Ardisia gigantifolia* revealed lower F_v_/F_m_ values in plants introduced into natural habitats than in individuals grown under controlled conditions. According to the authors, this reduction was attributed to the cumulative effects of multiple environmental stressors acting simultaneously under field conditions during the early phase of acclimatization. This was also confirmed by the results of studies conducted by Pogorzelec et al. [16] and Arciszewski et al. [42] on species of the genus *Salix* during the initial stage of plant growth in the natural environment. These studies indicated a significant intensification of physiological processes related to plant growth during acclimatization in substitute habitats, as evidenced by an improvement in the measured chlorophyll fluorescence parameter. In contrast, experiments conducted under laboratory conditions, involving the induction of stress using a single environmental factor (temperature, high salinity, or substrate eutrophication), revealed significantly smaller fluctuations in F_v_/F_m_ values in *S. lapponum* [43,44] than in the case of the combined effects of stress factors in substitute habitats during studies conducted in 2024–2025.

The fluctuations in the content of photosynthetic pigments (chlorophyll *a* and *b*) in the plant material of *S. lapponum* observed during the field experiment indicate adaptation of the photosynthetic apparatus to changing habitat conditions. At the reference site, the introduced plants exhibited a relatively constant level of pigment concentration in the examined leaf tissues during the entire period of the study. At the other experimental sites, changes in these values were observed, which may indicate unstable conditions and a potential, constant influence of stress factors of varying intensity in the substitute habitats.

During the analysis of photosynthetic pigment content in the tissues of introduced plants throughout the study period, particular attention was given to the Chl a/b ratio, which reflects structural and functional changes in the photosynthetic apparatus. A decrease in this parameter, especially in transformed and degraded habitats (D and E), indicated an increase in Chl *b* content, primarily associated with light-harvesting antenna complexes [45]. This phenomenon may be interpreted as an adaptive response to changing light conditions or as a result of photosynthetic apparatus reorganization in response to environmental stress [46,47].

Important insights into plant physiological status were also provided by the analysis of carotenoid content in plant tissues. These pigments play a key photoprotective role and are involved in the dissipation of excess excitation energy, thereby protecting the photosynthetic apparatus against oxidative stress [48,49]. An increase in the content of these pigments in plants at transformed habitats and in a peatland undergoing enhanced ecological succession may indicate the activation of protective mechanisms in response to less favorable environmental conditions. Similar relationships were also reported in conservation translocation studies conducted on other endangered species, including *Primulina* spp. and *Euryodendron excelsum*. These studies showed that individuals introduced to sites outside their natural range or translocated to microhabitats that did not fully meet the species’ specific ecological requirements were characterized by reduced photosynthetic efficiency, accompanied by lower photosynthetic pigment content [40,50]. These observations confirm the usefulness of studying changes in the levels of photosynthetic pigments as sensitive indicators of physiological stress and the effectiveness of acclimatization in plants introduced into new habitats.

Disruptions in photosynthesis may also result from disturbances in cellular water balance associated with a decline in relative water content (RWC). This parameter is considered one of the key indicators of plant water stress, reflecting the balance between water uptake and loss, as well as the hydration status of tissues [51]. The results obtained during the field experiment indicate a clear seasonal variation in RWC in the plant material of *S. lapponum* individuals at substitute sites, confirming the high sensitivity of this indicator to changes in habitat hydrological conditions. The greatest fluctuations in RWC in *S. lapponum* tissues were recorded during the summer months of 2024, particularly in degraded peatlands and raised bogs, whose functioning depends primarily on rainfall. This interpretation is supported by meteorological data from the nearest monitoring station in Włodawa (eastern Poland), which recorded exceptionally low precipitation in May 2024 (11.7 mm), followed by a gradual increase in June and July (unpublished data). Additional field observations also indicated temporary water deficits at the study sites, as physicochemical measurements of habitat water could not be performed at site D (drained peatland) in July 2024 and at sites B (raised bog) and D in August 2024, due to critically low groundwater levels (unpublished data). It should also be noted that the relative water content in tissues may depend on other habitat-related stress factors, such as excessive salinity or high nitrogen levels in the water available to plants [44].

Anthocyanins, classified as secondary plant metabolites, accumulate in tissues in response to various stress factors, such as low ambient temperature, water deficit, or excessive radiation [52,53]. Their content in the tissues of *S. lapponum* during the field experiment exhibited pronounced seasonal variability. However, no statistically significant differences in anthocyanin concentration were found among plants from different substitute sites. It can therefore be concluded that the plant response manifested, as anthocyanin accumulation is associated with environmental stress resulting from a combination of many abiotic factors acting seasonally at all substitute sites. The anthocyanin content in the fresh leaf biomass of *S. lapponum* at the experimental sites remained at a level similar to the values obtained in laboratory experiments, where acclimatized plants were subjected to stress associated with salinity and increased nitrogen supply [44]. Similar results were reported in studies on *Rhododendron minus*, where an increased accumulation of anthocyanins was observed in plants exposed to high levels of radiation. The authors suggest that this phenomenon was linked to photoprotective mechanisms that limit oxidative stress [54]. According to Naing and Kim [55], reactive oxygen species induced by abiotic stress may stimulate anthocyanin biosynthesis, which in turn may help to increase stress resistance by scavenging excess reactive oxygen species. Summing up, this indicator can be used to supplement the monitoring of the acclimatization of introduced plants. Its values should be interpreted in relation to other indicators, such as RWC or antioxidant enzyme activity.

Guaiacol peroxidase activity and the results of histochemical ROS detection indicate the response of plants to environmental stresses under various conditions in alternative habitats. In contrast to previous studies on *S. lapponum* conducted under controlled laboratory conditions, where plants were exposed to single stress factors such as salinity, eutrophication, or temperature extremes [43,44], the present field experiment revealed markedly higher guaiacol peroxidase activity, particularly in plants growing in transformed habitats such as drained and degraded peatlands. This suggests that under field conditions, stronger defense mechanisms are activated in response to multifactorial stress [56]. An increase in guaiacol peroxidase activity under environmental stress is a widely observed phenomenon, as demonstrated in studies on drought and salinity stress, where GPOX activity increased with the intensity of stressors, reflecting the activation of plant defense mechanisms associated with the neutralization of reactive oxygen species [57,58].

Under controlled laboratory conditions, histochemical detection of ROS in *S. lapponum* tissues exposed to single stress factors (low and high temperature, varying salinity levels, and increased nitrogen supply) [43,44] resulted in lower staining intensity than that observed in field experiments conducted in 2024–2025. These differences may result not only from the nature of the applied stress factors, but also from differences in the age and morphological traits of the studied plants. In the laboratory experiments, young individuals derived from ex situ cultivation were used, characterized, among other features, by a less developed indumentum layer on the leaf surface [43,44]. In contrast, plants introduced into natural sites exhibited well-developed, dense indumentum on thicker leaf blades. It should also be emphasized that the staining method is time-consuming and less standardized, so it can be used to confirm the presence of ROS detected by another, more sensitive method. As demonstrated by Grellet Bournonville and Díaz-Ricci [59], reliable quantitative determination of superoxides requires additional analytical steps, including extraction of the formazan and its spectrophotometric quantification.

The results of the study, together with an evaluation of the utility of individual biomarkers, enabled the identification of a set of indicators suitable for monitoring the outcomes of active conservation of the endangered relict species *S. lapponum*. These indicators are characterized by high sensitivity, functional relevance, and non-invasiveness, and their determination does not require advanced technical expertise. The values of these three indicators: maximum photochemical efficiency of photosystem II (F_v_/F_m_), relative water content (RWC), and guaiacol peroxidase activity, can support an objective assessment of the effectiveness of active protection measures based on the physiological responses of plants under natural conditions. However, it should be emphasized that the initial step in evaluating acclimatization success and identifying individuals of reduced vitality should be an expert-based assessment of plant morphological traits, subsequently complemented by more sensitive physiological indicators [60].

## 4. Materials and Methods

### 4.1. Plant Material and Experimental Sites

The plant material used in the experiment consisted of one-year-old *Salix lapponum* seedlings, introduced into five sites differing in terms of habitat conditions. The plants had previously been obtained through micropropagation from vegetative parts of parent plants [61] and acclimatized both in the laboratory and at the field plant acclimatization station located in close proximity to the planned introduction sites, where they grew for 2 months (according to the procedure described by Pogorzelec et al. [16]). After this period, 40 *S. lapponum* individuals were introduced to each experimental site in autumn 2023. The plants were manually planted with intact root balls into peat mats or directly into the soil, depending on habitat type. The seedlings were randomly distributed over an area of approximately 25 m^2^ at each site.

The sites where the plants were introduced (5 substitute habitats) were located in eastern Poland within the wetland complex of the Łęczna-Włodawa Lake District. This is an area where, as late as the 1950s, the presence of several numerous populations of the studied species was recorded [62]. Due to habitat transformation and fragmentation resulting from anthropogenic pressure, only a few remaining populations of *S. lapponum* were confirmed in this area after the year 2000 [15].

The substitute sites represented habitats intended to reflect potential transformations of peatland ecosystems under the influence of global warming (climate change) in the study area. A habitat exhibiting characteristics typical of transitional mires (site A) was designated as the reference site, as the largest currently existing population of *S. lapponum* in eastern Poland occurs under such conditions [15,63]. Additional introduction sites were identified during a field survey in the study area, in habitats that are both natural and unaffected by direct human activity (site B—a continental-type raised bog, whose proper functioning depends on rainfall; site C—a transitional mire undergoing accelerated ecological succession—as an example of a habitat whose abiotic environment is being modified by quantitative and qualitative changes in plant cover), as well as those that have been altered or degraded and can represent the effect of natural habitat transformation under the influence of climate warming (site D—a mesic meadow on peat soil, resulting from the drainage of peatland habitats—simulating the drainage of natural wetlands; site E—a degraded, desiccated transitional mire adjacent to a lake, resulting from the covering of the peatland surface with waste rock, representing a habitat permanently transformed by long-term fluctuations in groundwater levels and increased temperature).

### 4.2. Assessment of Physiological Parameters

The physiological parameters of the plants introduced into each substitute site were measured at monthly intervals during the growing season in the first year after planting (from May to September 2024) and at the beginning of the growing season in the second year (May and June 2025). At each sampling date, leaf material was collected from randomly selected individuals to ensure independence of observations.

#### 4.2.1. Selected Chlorophyll Fluorescence Parameters

Chlorophyll *a* fluorescence parameters, including minimal fluorescence (F_0_), maximal fluorescence (F_m_) and maximum quantum efficiency of photosystem II (F_v_/F_m_), were measured in situ on leaves located in the middle part of shoots of 10 randomly selected plants from each experimental population. Measurements were conducted using a field fluorometer (Handy PEA fluorometer, Hanstech Instruments, UK) following 15 min of dark adaptation.

#### 4.2.2. Photosynthetic Pigment Content

The content of chlorophyll *a*, chlorophyll *b*, and carotenoids was determined in leaves collected from the middle parts of the plant shoots. The plant material was homogenized in a 96% ethanol solution and then incubated in a water bath at 70 °C for 5 min to ensure complete extraction of the pigments. The samples were centrifuged (10,000 rpm, 10 min) to separate the solid phase from the liquid. The absorbance of the extracts was measured using a spectrophotometer (SPECORD 40, Analytik Jena, GmbH, Jena, Germany) at wavelengths of 470, 649, and 665 nm. Each sample was analyzed in three technical replicates. Pigment concentration was calculated using a modified method of Lichtenthaler and Welburn [64] as follows:

Chl *a* = (13.95 × A_665_) − (6.88 × A_649_) (1)

Chl *b* = (24.96 × A_649_) − (8.12 × A_665_) (2)

Car = ((1000 × A_470_) − (2.05 × Chl *a*) − (114.8 × Chl *b*))/245 (3)

#### 4.2.3. Anthocyanin Content

The anthocyanin content was determined according to the method described by Martinez and Favret [65]. Leaf blade fragments (100 mg, composite sample; each composite sample consisted of 3 plants) were extracted in methanol acidified with HCl (99:1, *v*/*v*) for 24 h at 4 °C. The samples were centrifuged (10,000 rpm, 10 min), after which the absorbance of the supernatant was measured spectrophotometrically at wavelengths of 527 and 652 nm, with each biological sample measured in three technical replicates.

#### 4.2.4. Relative Water Content

Relative water content (RWC) was determined according to the method of González and González-Vilar [66]. Five leaves collected from randomly selected individual plants were used for the analysis, and their fresh weight (FW) was determined. The samples were then placed in distilled water for 24 h to achieve full turgor and were weighed again (TW). In the final stage, the leaf blades were dried at 60 °C for 24 h in a drying oven (SML 32/250, Zalmed, Warsaw, Poland), and the dry weight (DW) was determined. The RWC index was calculated using the formula:

RWC = (FW − DW)/(TW − DW) × 100%.(4)

#### 4.2.5. Guaiacol Peroxidase Activity

To determine guaiacol peroxidase activity (GPOX, EC 1.11.1.7), 100 mg of leaf material (composite sample; each composite sample consisted of three plants) was used and homogenized in 1 mL of 0.05 M phosphate buffer (pH 7.0). The obtained homogenates were centrifuged for 10 min (10,000 rpm) at 4 °C. Enzyme activity was determined spectrophotometrically (SPECORD 40 spectrophotometer, Analytik Jena GmbH, Jena, Germany). GPOX activity was assayed according to the method of Małoplesza and Urbanek [67]. Changes in absorbance were recorded at 480 nm for 4 min, with readings taken at 1-min intervals. Each biological sample was analyzed in three technical replicates.

#### 4.2.6. Histochemical Detection of Reactive Oxygen Species (ROS)

Qualitative analyses of hydrogen peroxide (H_2_O_2_) and superoxide anion (O_2_^−^) accumulation in leaf tissues were conducted in spring and summer 2024, during the first year following translocation. For each experimental site, four leaves collected from randomly selected plants were used for histochemical staining. The analyses followed the method described by Kumar et al. [68]. Reactive oxygen species were detected using 3,3′-diaminobenzidine (DAB) for H_2_O_2_ and nitro blue tetrazolium chloride (NBT) for O_2_^•−^. Leaves were incubated for 12 h in darkness at room temperature in previously prepared solutions of DAB (1 mg mL^−1^) and 0.2% NBT in 50 mM phosphate buffer (pH 7.5). After incubation, the leaves were immersed in hot water and subsequently decolorized in hot ethanol to remove chlorophyll. As a result of the reaction, the presence of H_2_O_2_ led to the formation of a brown DAB polymerization product, whereas the reduction of NBT resulted in the formation of a blue–purple diformazan, indicating the presence of O_2_^•−^.

### 4.3. Statistical Analysis

The normality of data distribution was tested using the Shapiro–Wilk test. Since most of the analyzed variables did not meet the assumptions of normal distribution, further analyses were performed using non-parametric statistics. Differences in physiological parameters among the experimental sites within individual sampling terms were assessed using the Kruskal–Wallis test (significance level *p* ≤ 0.05). The same test was also applied to evaluate temporal changes within individual sites across consecutive sampling periods. All statistical analyses and data visualizations were performed using the R statistical environment.

## 5. Conclusions

This study constitutes the first attempt to identify physiological indicators that may enhance monitoring the condition of *Salix lapponum* plants following translocation into substitute habitats representing different stages of habitat transformation. Analysis of the results suggests that the most effective approach is the combined use of parameters reflecting photosynthetic performance, plant water status, and the level of antioxidant response to environmental stress. Among the parameters evaluated in this study, the most informative biomarkers for post-translocation monitoring of *S. lapponum* were maximum photochemical efficiency of photosystem II (F_v_/F_m_), relative water content (RWC), and guaiacol peroxidase activity. At the same time, the analysis of physiological parameters should be conducted in parallel with observations of plant morphological traits.

## Data Availability

The original contributions presented in this study are included in the article. Further inquiries can be directed to the corresponding author.

## Abbreviations

The following abbreviations are used in this manuscript:

ACNs	Anthocyanins
GPOX	Guaiacol Peroxidase
ROS	Reactive Oxygen Species
RWC	Relative Water Content
PSII	Photosystem II
